# Race and Regional Differences in Cerebral Microbleeds in Individuals with Incident Stroke or Transient Ischemic Attack: The REGARDS Study

**DOI:** 10.3390/brainsci16090934

**Published:** 2026-08-31

**Authors:** Oluchi Ekenze, Liang Niu, Russell P. Sawyer, Aleena Bennet, Mary Cushman, Vasileios-Arsenios Lioutas, Hugo J. Aparicio, Sudha Seshadri, Virginia J. Howard, Jose R. Romero, Hyacinth I. Hyacinth

**Affiliations:** 1 Framingham Heart Study, Framingham, MA 01702, USA; osekenze@bu.edu (O.E.); vlioutas@bidmc.harvard.edu (V.-A.L.); hugoa@bu.edu (H.J.A.); seshadri@uthscsa.edu (S.S.);; 2 Department of Neurology, Boston University Chobanian & Avedisian School of Medicine, Boston, MA 02118, USA; 3 Department of Biostatistics, Health Informatics, and Data Sciences, University of Cincinnati, Cincinnati, OH 45267, USA; niulg@ucmail.uc.edu; 4 Department of Neurology & Rehabilitation Medicine, College of Medicine, University of Cincinnati, Cincinnati, OH 45267, USA; sawyerrl@ucmail.uc.edu; 5 Department of Biostatistics, School of Public Health, The University of Alabama at Birmingham, Birmingham, AL 35233, USA; amosher@uab.edu; 6 Department of Medicine, Larner College of Medicine, University of Vermont, Burlington, VT 05401, USA; 7 Department of Neurology, Beth Israel Deaconess Medical Center, Harvard Medical School, Boston, MA 02215, USA; 8 The Glenn Biggs Institute for Alzheimer’s and Neurodegenerative Diseases, University of Texas Health Sciences Center, San Antonio, TX 78229, USA; 9 Department of Epidemiology, School of Public Health, The University of Alabama at Birmingham, Birmingham, AL 35233, USA; vjhoward@uab.edu

**Keywords:** black persons, cerebral microbleeds, non-stroke belt, stroke belt, white participants

## Abstract

**Background**: Cerebral microbleeds (CMBs) are associated with risk of stroke, cognitive impairment, and dementia. The influence of race and/or region of residence on CMB is underexplored. **Methods**: REasons for Geographic and Racial Differences in Stroke (REGARDS) study participants with incident stroke/TIA and available MRI were included. CMBs were classified as any, non-lobar, lobar or mixed CMB. Counts were recorded. Multivariable logistic regression assessed associations between race and/or region of residence with CMB presence. Multivariable negative binomial regression related these factors to CMB counts. Models adjusted for age, sex, vascular risk factors. **Results**: Among 808 participants (317 Black participants, 412 in stroke belt), Black participants had higher odds of any CMB, odds ratio (OR) = 1.72; 95% confidence interval (CI) 1.22, 2.43). Stroke belt residents had lower odds of any (OR = 0.51, 95% CI 0.37, 0.72), non-lobar (OR = 0.46; 95% CI 0.24, 0.85), lobar (OR = 0.60; 95% CI 0.38, 0.93) CMBs and lower odds of CMB counts (rate ratio [RR] = 0.66, 95% CI 0.43, 0.99). Black participants residing in the stroke belt had higher odds of any (OR = 1.88; 95% CI 1.11, 3.20) and mixed (OR = 2.99; 95% CI 1.19, 7.74) CMBs. **Conclusions**: Black participants had higher risk for CMBs. Higher risk in Black participants may suggest increased subclinical vascular brain injury.

## 1. Introduction

Cerebral microbleeds (CMBs) are subclinical markers of vascular brain injury, reflecting underlying deposits of hemosiderin, and associated with a higher risk of cognitive impairment, dementia, Alzheimer’s disease, stroke and stroke recurrence [1,2,3]. CMBs arise from vessel wall damage most frequently caused by hypertensive arteriopathy or cerebral amyloid angiopathy, depending on their predominant brain location [2]. Lobar CMBs are more commonly seen in individuals with cerebral amyloid angiopathy or advanced hypertensive arteriopathy whereas deep CMBs are more commonly seen in individuals with hypertensive arteriopathy [4]. The clinical relevance of CMBs is highlighted by the fact that they are associated with a higher risk of cerebral hemorrhagic complications in individuals receiving anticoagulants for stroke prevention or thrombolytic therapy for stroke treatment.

Prior research has shown racial and regional differences in stroke and dementia, with Black and Hispanic individuals having higher incidence and prevalence of stroke [5,6], and US stroke belt residents having a higher incidence of stroke and mortality from stroke [7]. Black individuals also have higher prevalence of vascular risk factors, and are less likely to achieve control of stroke risk factors such as hypertension [8,9,10].

Although many studies have addressed the relationship of demographic and vascular risk factors with CMB prevalence and burden [1,11,12,13,14], there is paucity of data from studies that include a substantial number of Black participants, or that examines the burden of CMBs in the stroke belt. Understanding how race and/or region of residence affect the distribution of the burden of CMBs, its brain topological distribution especially in a population (with stroke/Transient Ischemic Attack (TIA)) with the highest risk for cerebrovascular diseases, may provide essential insight that could guide the development of preventive efforts. Our hypothesis is that among participants with stroke or TIA, Black participants and residents in the stroke belt region of the US will have a higher prevalence and burden of CMBs.

## 2. Material and Methods

### 2.1. Sample

The study sample was derived from the REasons for Geographic And Racial Differences in Stroke (REGARDS) study, a national longitudinal cohort study of 30,239 English-speaking, community dwelling individuals enrolled in 2003–2007. Participants described themselves as either Non-Hispanic Black (hereafter referred to as Black participants) or Non-Hispanic White (hereafter referred to as White participants), were 45–81 years old at time of enrollment and resided within the contiguous 48 states of the USA [15]. REGARDS enrolled 42% Black participants and 56% of participants resided in the stroke belt of the USA, which includes eight states of North Carolina, South Carolina, Georgia, Tennessee, Mississippi, Alabama, Louisiana, and Arkansas [15]. The stroke belt region of the USA has a high prevalence of stroke [7,16] and stroke mortality [7] probably due larger proportion of Blacks, and residents with higher prevalence of traditional stroke risk factors, lower socioeconomic status, less access to care, and larger proportion of rural residents [7].

At enrollment, participants underwent a computer-assisted telephone interview (CATI) by a trained interviewer to obtain demographics, medical history and lifestyle factors. This was followed 3–4 weeks later by an in-person assessment of risk factors (blood pressure, anthropometrics, and electrocardiogram), and collection of blood and urine samples which were sent overnight to a central laboratory [15]. Participants have been followed at 6-month intervals by CATI to identify suspected stroke events and obtain cognitive assessments. For suspected strokes, medical records including neuroimaging and other diagnostic reports are retrieved and adjudicated by stroke experts.

Written informed consent was obtained during the in-person visit and institutional review boards of participating institutions approved the study methods. Details of the study design and stroke adjudication processes are provided elsewhere [5,15].

### 2.2. Brain Magnetic Resonance Imaging (MRI) Acquisition

For the confirmed stroke and TIA cases, the MRIs scans associated with the event and done at the time of stroke/TIA were requested and used for the assessment of CMB. The approach for retrieving and handling these MRI scans and relevant quality control measures have been published [17]. Briefly, participants or their proxies are asked the name of the facility where they received most of their evaluation and treatment. Medical records, including radiology reports from MRIs and/or computerized tomography scans, are then requested. Based on receipt of the MRI report, requests for the MRIs scan files are mailed to the medical facility. Retrieved MRIs are submitted to the images analysis team for ratings of various cerebral small vessel disease (CSVD) markers using established criteria [18].

This report included participants who self-reported as stroke-free at enrollment, had either an adjudicated stroke (ischemic or hemorrhagic) or TIA after enrollment, during the follow-up period (2003–2022), and had available MRI for CMB ratings.

### 2.3. Exposure

The exposure in this study was self-identified race/ethnicity (Non-Hispanic Black vs. None-Hispanic White) and region of residence at enrollment (stroke belt vs. non-stroke belt).

### 2.4. Outcome

The primary outcome is any CMB described as presence of at least one CMB in any brain region. CMBs were identified using published guidelines, as rounded or ovoid hypointense lesions less than 10 mm in diameter and surrounded by brain parenchyma over at least half of the circumference on a T2*-gradient recalled/repeat echo weighted (T2-GRE) sequence or susceptibility-weighted imaging (SWI) [2,18]. CMBs were rated by 3 neurologists (O.E, J.R.R, H.I.H.) who have vast experience in CMB ratings, and were blinded to the participants clinical or demographic data, with excellent inter-rater (Kappa statistics 0.87–0.90) and intra-rater reliability (Kappa 0.87). CMB presence was grouped according to brain topography into 3 categories: non-lobar CMB only (deep including infratentorial CMB), lobar CMB only, based on presence of at least one CMB in each brain topography and mixed CMB (non-lobar and lobar CMB). CMB count was also recorded. The brain topography of CMB (non-lobar only, lobar only and mixed) and total count of CMB (i.e., CMB burden) was evaluated as secondary outcomes.

### 2.5. Covariates

Covariates were obtained from the baseline assessment 2003–2007. Hypertension was defined according to the Seventh Report of the Joint National Committee on Prevention, Detection, Evaluation of High blood pressure (JNC 7) criteria used at that time as systolic blood pressure ≥ 140 mmHg, diastolic blood pressure ≥ 90 mmHg or use of antihypertensive medications [19]. Diabetes was defined as a non-fasting blood glucose ≥ 200 mg/dL (≥11.1 mmol/L), fasting glucose ≥ 126 mg/dL (≥7 mmol/L) or self-reported use of insulin or oral hypoglycemic medications. Total cholesterol (mg/dL) was measured on fasting specimens. Self-reported smoking was used to define the smoking status and classified as current (within the past year) past smoker, or never. Missingness of covariates was less than 1%.

### 2.6. Statistical Analysis

Descriptive statistics for clinical and demographic characteristics included mean and standard deviation mean (SD) for continuous variables and counts and percentages for categorical variables. Multivariable logistic regression analysis was used to determine the odds ratio and 95% confidence interval (CI) for the association between race and/or region of residence with presence of CMB. In exploration analysis, we evaluated associations between race and/or region of residence with brain topography of CMB—non-lobar CMB only, lobar CMB only, mixed CMB using multivariable logistic regression. Multivariable negative binomial regression was used to determine rate ratios and 95% CI for the relationship between race and/or region of residence and CMB counts.

For all analysis, we evaluated the effect of race within each region and the effect of region within each race. Models were adjusted for age and sex (model 1) and additionally vascular risk factors: hypertension, diabetes, smoking, total cholesterol (model 2). A *p*-value < 0.05 was considered statistically significant. We also assessed the multiplicative interaction of race and region of residence with any, non-lobar and lobar CMB. A threshold of *p*-value < 0.1 was used to identify significant interaction. All analysis was performed using R statistical software version 4.1.2.

## 3. Results

### 3.1. Subjects

**Figure 1:**
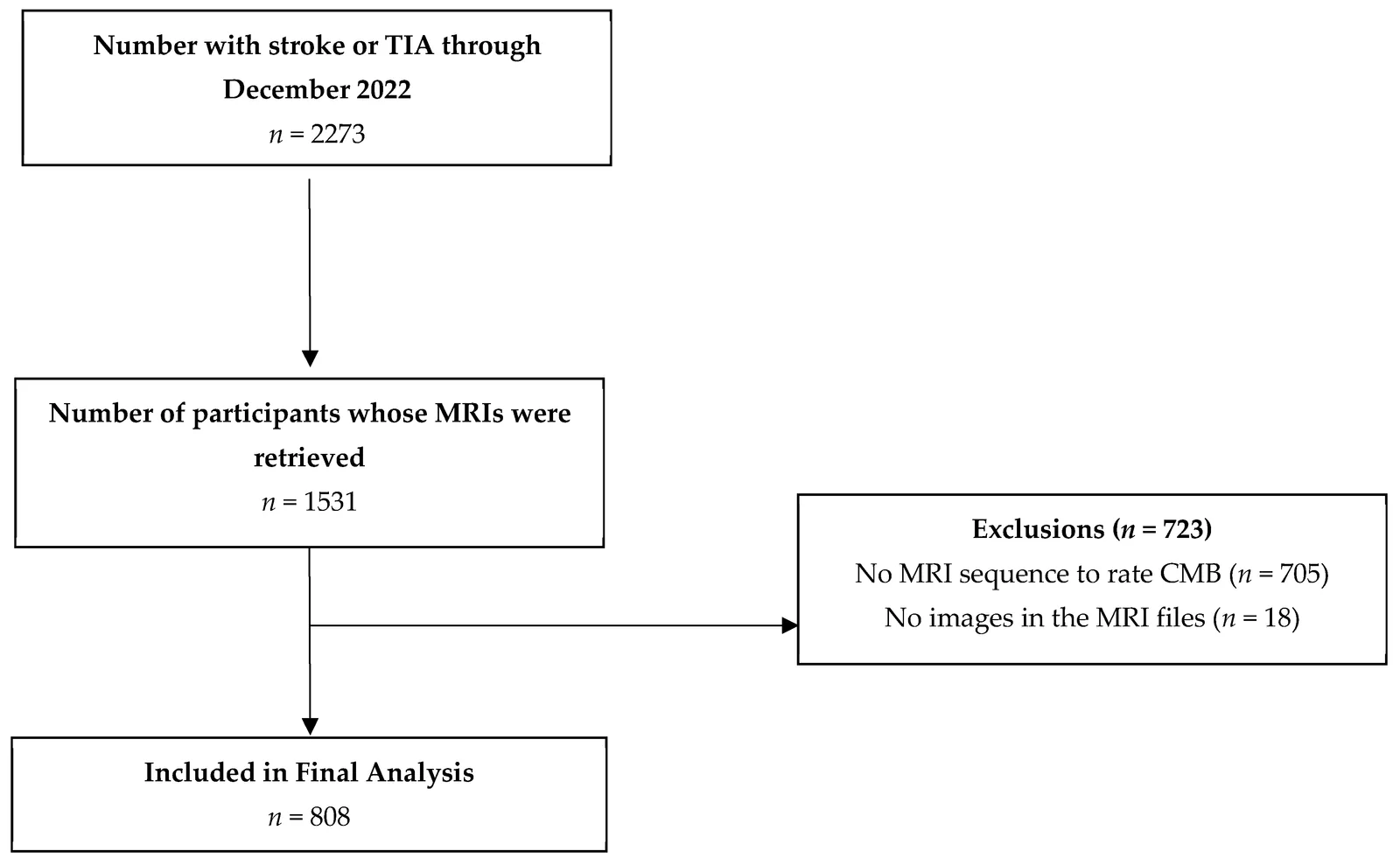
Flow chart of sample selection.

In total, there were 2273 participants with incident stroke or TIA through December 2022, and we were able to retrieve the MRIs from 1531 participants (Figure 1). Retrieval and ratings of MRI are still ongoing. Of the 1531 participants with available MRI, 808 (mean age 67 ± 8 years, 51% female, 39.2% Black) participants had a T2-GRE or SWI MRI sequence allowing for CMB ratings (699 and 109 CMBs rated using T2-GRE and SWI respectively) and were included in our analysis. Participants who were missing the above MRI sequences were excluded. Excluded participants were slightly older (69 ± 9 vs. 67 ± 8 years) and had a slightly higher proportion with hypertension (70% versus 64%). For two participants, only prevalence/non-prevalence of any CMB could be ascertained and they were not included in brain topography analysis. Other characteristics and risk factors profile were similar between the two groups (Table 1, Supplementary Table S2).

### 3.2. CMB Prevalence and Topography by Race and Region of Residence

In Supplementary Table S1, the prevalence of CMBs (i.e., any CMB or having CMB in any brain region) was 26% overall; 6% had non-lobar CMB only, 13% had lobar CMB only, and 7% had mixed (i.e., non-lobar + lobar). More Black participants had any CMB than White participants (32% vs. 23%), a pattern similar for non-lobar only (8% vs. 5%), lobar only (15% vs. 11%) and mixed CMBs (8.5% vs. 6.5%). Residents of the stroke belt had lower prevalence of CMBs than those residing elsewhere; any CMBs (20% vs. 33%), non-lobar (4% vs. 9%), lobar (10% vs. 16%) and mixed (6% vs. 9%) CMBs.

### 3.3. CMBs and Risk Factor Levels

Among Black participants who had any CMB, hypertension occurred more in those residing in the non-stroke belt compared to those residing in the stroke belt (81% versus 76%). Among White participants who had any CMB, those residing in the non-stroke belt had slightly lower proportion with hypertension compared to stroke belt dwellers (63% versus 65%). The rest of the results of additional vascular risk factor comparison is presented in Table 2.

#### Multivariable Analyses of the Association of Race and Geographic Region with CMB Overall and in Different Regions

Associations of race and region with CMB overall and by brain regions are shown in Table 3.

### 3.4. Primary Outcome

#### Any CMB

In fully adjusted multivariable models (Table 3), irrespective of region of residence at baseline, Black participants had a significantly higher odds for any CMB (OR = 1.72, 95% CI = 1.22–2.43). Residents of the stroke belt region irrespective of race had lower odds for having any CMB (OR = 0.51; 95% CI = 0.37–0.72). Considering only residents of the stroke belt, Black participants were more likely than White participants to have any CMBs (OR = 1.88; 95% CI = 1.11–3.20). Estimates remained unchanged after additional adjustments for MRI sequence (Supplementary Table S3). There was no significant interaction between race and region on risk of having CMB (*p* for interaction = 0.95).

### 3.5. Secondary Outcomes

#### 3.5.1. Non-Lobar CMB Only

In the overall sample, there were no significant differences in non-lobar CMB by race, but stroke belt residence was associated with lower odds of non-lobar CMB (OR = 0.46; 95% CI = 0.24–0.85). The odds of non-lobar CMB were not significantly higher in Black compared to White participants residing in the stroke belt (OR = 1.75; 95% CI = 0.60–5.12) or non-stroke belt (OR = 1.21; 95% CI = 0.54–2.65) (Table 3). Estimates remained unchanged after additional adjustments for MRI sequence (Supplementary Table S3). There was also no significant interaction between race and region on the risk of having only non-lobar CMB (*p* for interaction = 0.72).

#### 3.5.2. Lobar CMB Only

Like non-lobar CMB, there was no significant association of race with having only lobar CMB. Participants residing in the stroke belt had lower odds of lobar CMB (OR = 0.60; 95% CI = 0.38–0.93) (Table 3) and there was no significant interaction between race and region on the risk of having only lobar CMB (*p* for interaction = 0.33). Estimates remained unchanged after additional adjustments for MRI sequence (Supplementary Table S3).

#### 3.5.3. Mixed CMB

In the overall sample, there was no association of race or region with presence of mixed CMB. Black participants residing in the stroke belt were more likely to have mixed CMB (OR = 2.99, 95% CI = 1.19–7.74) compared with White participants residing there (Table 3). Estimates remained unchanged after additional adjustments for MRI sequence (Supplementary Table S3). However, there was no significant interaction between race and region on the risk of having mixed CMB (*p* for interaction—0.21).

#### 3.5.4. CMB Counts

Black participants did not have a higher CMB count than White participants (RR of 1 higher CMB = 1.37; 95% CI = 0.88–2.12). Participants residing in the stroke belt had a significantly lower likelihood of a higher CMB count (RR = 0.66; 95% CI = 0.43–0.99). White participants residing in the stroke belt had lower risk of a higher CMB count (RR 0.46; 95% CI = 0.26–0.84) while Black participants residing there had higher risk (RR 1.85; 95% CI = 0.91–3.80), though not significant (*p* interaction 0.16). Outside the stroke belt there was no association of race with higher CMB count (RR 1.27; 95% CI 0.76–2.13 for Black compared to White participants). Estimates remained unchanged with further adjustment for MRI sequence (Supplementary Table S3).

## 4. Discussion

Among 808 REGARDS study participants with stroke or TIA and MRI data, the prevalence of any CMB was 26%. Racial differences in CMB prevalence were also noted, with Black participants having higher odds for any CMB, but not CMB in specific regions or CMB count. Unexpectedly, participants residing in the stroke belt had lower odds for any CMB, non-lobar, lobar CMB and less likely to have higher CMB count.

The prevalence of CMB in our study was comparable to prior studies of persons with stroke [12,20,21,22] and those without stroke [11,13,14,22]. However, the participants with CMB in our study were younger than in some previous reports in people who had stroke (mean age of participants with CMB was 68 years in our study versus 70 years [20], 77.1 years [21], 78.5 years [22] and 85.2 years [12] in prior studies) suggesting that vascular brain injury may occur at a relatively younger age in our cohort.

We observed that Blacks participants had higher odds for any CMB, a finding that differed from prior studies from the Washington Heights/Inwood Columbia Aging Project [12] and the Northern Manhattan study (NOMAS) [14]. This contrast may relate to differences between the populations studied. Our study had a larger proportion of Black participants (317/808 [39.2%] versus 138/935 [15%] [14] and 86/243 [35.4%] [12]) and included participants living in the stroke belt and non-stroke belt region of the USA. Our findings add to existing knowledge since prior studies on CMB included predominantly non-stroke belt dwellers with stroke [12,20,21,22] or from population-based studies in people without stroke [1,11,14].

We observed that stroke belt residence was associated with a lower risk of any CMB, lobar and non-lobar CMB, and lower CMB count. This was unexpected given the high prevalence of hypertension and other stroke related risk factors in this region. It is possible that this observation relates to the lower baseline age of residents in the stroke belt compared to outside the stroke belt, among this group of people with available MRIs. In addition, other factors such as high stroke mortality in the stroke belt, less access to care in a primary stroke center among stroke belt residents or residual confounding may account for the observation [7]. However, Black participants living in the stroke belt had higher risk of having any CMB and mixed CMB compared to White participants residing in the stroke belt. The pattern was the same for Black participants residing outside the stroke belt, though not statistically significant, so caution in interpretation is warranted and more research is needed. The higher prevalence of hypertension, or its greater severity and duration among the Black participants, might be partly responsible for this observation. Hypertension may lead to cerebral small vessel disease including CMB through multiple interconnected mechanisms including endothelial dysfunction [23,24] and vascular inflammation [24]. Further, prior REGARDS studies showed that compared to Whites, Black participants living in the stroke belt have higher prevalence and severity of hypertension, are more likely to be on more than one medication for hypertension, and are less likely to have controlled blood pressure [25,26]. Taken together, our finding may suggest that higher CMB prevalence among Black participants may reflect cumulative and incompletely measured vascular and social determinants, including hypertension severity and duration, treatment access, blood pressure control, socioeconomic factors, and structural inequities.

We evaluated the topography of CMB because lobar CMB is commonly associated to cerebral amyloid angiopathy (CAA) while non-lobar CMB is associated with hypertensive arteriopathy [2,4]. Our findings of a higher risk of any and mixed CMB in Blacks participant living in the stroke belt compared to White participants may suggest the possibility that both hypertensive arteriopathy and higher risk of CAA may be responsible for CMB in Black participants in the stroke belt. However, this requires additional imaging and or pathological studies for confirmation of the presence of mixed etiology and should be viewed as exploratory. Additionally, since there was no interaction of race and region of residence, it is possible that the difference in risk of any CMB and mixed CMB between Black and White participants in the stroke belt may not be due to the region of residence but rather other risk factors/persistent disparity that may be clustered and requires further exploration.

Strengths of our study include the bi-racial composition, the long follow-up, the novel evaluation of the influence of region of residence (stroke belt versus non-stroke belt) on the burden and risk of CMB, and the blinded CMB rating with high reproducibility. Limitations are that participants were included based on availability of MRI scans done for evaluation of incident stroke or TIA, thus selection bias needs to be considered. Also, due to small sample size in participants with non-lobar and mixed CMB, our analysis should be interpreted with caution and viewed as hypothesis generating. Since we used baseline age of the participants in analysis, our finding that stroke belt residents had any and lobar CMB at a younger age may likely be due to the younger age at baseline for participants who live in the stroke belt compared to those residing in the non-stroke belt. The excluded participants who were missing MRI were older and more likely to have hypertension; thus, the true burden of CMB could be higher than we observed which may have biased some of our results towards the null. Further, region of residence was based on location at the time of enrollment without considering the length of exposure and age at the time of exposure to the stroke belt. A prior REGARDS study [27] observed differences in stroke risk in participants in the stroke belt depending on length of time living in the stroke belt, such that those who lived their entire life in the stroke belt or had high proportion of residency in the stroke belt between ages 0–12 years had highest risk for stroke. Lastly, due to the small sample size, unavailability of possible confounders like antithrombotic and anticoagulants, stroke subtype, and covariates measured at baseline which may differ from assessment at the time of CMB evaluation, the interpretation of the results, especially for interaction testing, requires caution. Larger studies are needed to properly address the questions raised by some of our findings.

## 5. Conclusions

In this study of participants with an incident stroke or TIA, Black participants had higher prevalence of CMB. Although stroke belt residency was related to a lower risk of CMB and lower CMB count, this was only clearly seen among White participants. These findings highlight differences in prevalence of advanced subclinical vascular brain injury that might be seen between racial groups and geographic regions in the US. Additionally, irrespective of residency, Black participants had a higher risk for CMBs compared with White participants. Region of residency was not associated with CMB prevalence or number among Black participants. Further studies are needed to elucidate the underlying factors including potential differences in genetic and environmental exposures that influence CMB epidemiology, to enable development of effective and targeted preventative strategies to mitigate the adverse consequences of CMBs.

## Figures and Tables

**Table 1:** Descriptives characteristics of study participants and those excluded.

Variables		All Participants with Incident Stroke or TIA, *n* = 2273									
		Included *n* = 808					Excluded *n* = 1465				
		Stroke Belt Residence *n* = 412		Non-Stroke Belt Residence *n* = 396			Stroke Belt Residence *n* = 820		Non-Stroke Belt Residence *n* = 645		
	All Included	Black Participants *n* = 158	White Participants *n* = 254	Black Participants *n* = 159	White Participants *n* = 237	All Excluded	Black Participants *n* = 269	White Participants *n* = 551	Black Participants *n* = 295	White Participants *n* = 350	*p* Values for All Included and Excluded
Age in years, mean (SD)	67 (8)	64 (8)	66 (8)	68 (8)	68 (8)	69 (9)	66 (9)	70 (9)	68 (9)	71 (9)	<0.001
Men, *n* (%)	397 (49)	61 (39)	120 (47)	67 (42)	149 (63)	713 (49)	101 (38)	288 (52)	115 (39)	209 (60)	0.866
Women, *n* (%)	411 (51)	97 (62)	134 (53)	92 (58)	88 (37)	752 (51)	168 (62)	263 (48)	180 (61)	141 (40)	
Hypertension, *n* (%) ^#ԑ^											0.003
Yes	515 (64)	116 (73)	147 (58)	125 (79)	127 (53.6)	1024 (70)	223 (83)	349 (63)	237 (80.4)	215 (61.4)	
No	291 (36)	41 (26)	107 (42)	34 (21)	109 (46)	436 (30)	46 (17)	199 (36)	57 (19.3)	134 (38.3)	
Smoking status ^ԑ^											0.062
Past	337 (42)	60 (38)	99 (39)	59 (37)	119 (50.2)	647 (44)	106 (39)	241 (44)	125 (42.4)	175 (50)	
Current	97 (12)	33 (21)	25 (10)	21 (13)	18 (7.6)	208 (14)	42 (16)	82 (15)	54 (18.3)	30 (9)	
Never	373 (46)	65 (41)	130 (51)	79 (50)	99 (41.8)	604 (41)	119 (44)	227 (41)	115 (39)	143 (41)	
Diabetes ^ԑ^											0.11
yes	212 (26)	60 (38)	57 (22.5)	56 (35.2)	39 (16)	432 (30)	123 (46)	127 (23)	99 (33)	83 (23.7)	
no	594 (74)	98 (62)	196 (77)	102 (64.2)	198 (84)	1028 (70)	146 (54)	422 (76.6)	194 (66)	266 (76)	
Total cholesterol, mean (SD)	195 (42)	196 (45)	195 (41)	196 (44)	194 (38)	191 (40)	191 (45)	189 (40)	197 (39)	187 (38)	0.015

^#^ missing 1% in some cells, ^ԑ^ missing < 1% in some cells.

**Table 2:** Descriptive characteristics according to CMB topography and race in stroke belt region and non-stroke belt regions.

	Stroke Belt								Non-Stroke Belt							
Variables	Black Participants *n* (%) = 158 (38)								Black Participants *n* (%) = 159 (40)							
	Any CMB		Non-Lobar CMB		Lobar CMB		Mixed CMB		Any CMB		Non-Lobar CMB		Lobar CMB		Mixed CMB	
	No *n* = 120 (76)	Yes *n* = 38 (24)	No *n* = 149 (95)	Yes *n* = 8 (5)	No *n* = 141 (90)	Yes *n* = 16 (10)	No *n* = 144 (92)	Yes *n* = 13 (8)	No *n* = 97 (61)	Yes *n* = 62 (39)	No *n* = 143 (90)	Yes *n* = 16 (10)	No *n* = 127 (80)	Yes *n* = 32 (20)	No *n* = 145 (91)	Yes *n* = 14 (9)
Age, mean (SD)	63 (8)	65 (8)	64 (8)	64(10)	63 (8)	65 (7)	64 (8)	65(8)	67 (8)	69 (8)	67 (8)	71 (8)	67 (8)	68 (8)	68 (8)	66 (7)
Men, *n* (%)	46 (38)	15 (39)	57 (38)	3 (37.5)	55 (39)	5 (31)	54 (37.5)	6 (46)	42 (43)	25 (40)	62 (43)	5 (31)	55 (43)	12 (37.5)	59 (41)	8 (57)
Women, *n* (%)	74 (62)	23 (61)	92 (62)	5 (62.5)	86 (61)	11 (69)	90 (62.5)	7 (54)	55 (57)	37 (60)	81 (57)	11 (69)	72 (57)	20 (62.5)	86 (59)	6 (43)
Hypertension **^#^**^ԑ^, *n* (%)																
Yes	87 (73)	29 (76)	108 (72)	7 (87.5)	104 (74)	11 (69)	105 (73)	10 (77)	65 (67)	50 (81)	112 (78)	13 (81)	99 (78)	26 (81)	114 (79)	11 (79)
No	32 (27)	9 (24)	40 (27)	1 (12.5)	36 (25)	5 (31)	38 (26)	3 (23)	32 (33)	12 (19)	31 (22)	3 (19)	28 (22)	6 (19)	31 (21)	3 (21)
Smoking status ^#ԑ^, *n* (%)																
Past smokers	46 (38)	14 (37)	58 (39)	2 (25)	54 (38)	6 (37.5)	54 (37)	6 (46)	36 (37)	23 (37)	50 (35)	9 (56)	49 (38)	10 (31)	55 (38)	4 (29)
Never smokers	47 (39)	18 (47)	60 (40)	4 (50)	56 (40)	8 (50)	59 (41)	5 (38)	52 (54)	27 (44)	75 (52)	4 (25)	63 (50)	16 (50)	72 (50)	7 (50)
Current smokers	27 (23)	6 (16)	31 (21)	2 (25)	31 (22)	2 (12.5)	31 (22)	2 (16)	9 (9)	12 (19)	18 (13)	3 (19)	15 (12)	6 (19)	18 (12)	3 (21)
Diabetes ^#ԑ^, *n* (%)																
Yes	48 (40)	12 (32)	57 (38)	3 (37.5)	53 (38)	7 (44)	58(40)	2 (15)	35 (36)	21 (34)	50 (35)	6 (37.5)	46 (36)	10 (31)	51 (35)	5 (36)
No	72 (60)	26 (68)	92 (62)	5 (62.5)	88 (62)	9 (56)	86 (60)	11 (85)	61 (63)	41 (66)	92 (64)	10 (62.5)	80 (63)	22 (69)	93 (64)	9 (64)
Total cholesterol, mean (SD)	199 (47)	189 (40)	197 (46)	173 (32)	197 (46)	191(39)	196 (45)	194 (47)	195 (42)	196 (48)	195 (41)	205 (67)	196 (45)	194 (44)	196 (45)	193 (35)
	**White Participants *n* (%) = 254 (62)**								**White Participants *n* (%) = 237 (60)**							
	**Any CMB**		**Non-Lobar CMB**		**Lobar CMB**		**Mixed CMB**		**Any CMB**		**Non-Lobar CMB**		**Lobar CMB**		**Mixed CMB**	
	**No** ***n*** ** = 211 (83)**	**Yes** ***n*** ** = 43 (17)**	**No** ***n*** ** = 246 (97)**	**Yes** ***n*** ** = 8** **(3)**	**No** ***n*** ** = 230** **(91)**	**Yes** ***n* = 24** **(9)**	**No** ***n*** ** = 243 (96)**	**Yes** ***n*** ** = 11 (4)**	**No** ***n*** ** = 167** **(70)**	**Yes** ***n*** ** = 70** **(30)**	**No** ***n*** ** = 218 (92)**	**Yes** ***n*** ** = 18 (8)**	**No** ***n*** ** = 205 (87)**	**Yes** ***n*** ** = 31 (13)**	**No** ***n*** ** = 215 (91)**	**Yes** ***n*** ** = 21 (9)**
Age, mean (SD)	66(8)	67 (7)	66 (8)	68 (7)	66 (8)	67 (7)	66 (8)	68 (7)	68 (9)	70 (7)	68 (8)	69 (7)	68 (9)	71 (7)	68 (9)	69 (8)
Men, *n* (%)	97 (46)	23 (53)	115 (47)	5 (62.5)	109 (47)	11 (46)	113 (46.5)	7 (64)	108 (65)	41 (59)	142 (65)	7 (39)	125 (61)	24 (77)	139 (65)	10 (48)
Women, *n* (%)	114 (54)	20 (47)	131 (53)	3 (37.5)	121 (53)	13 (54)	130 (53.5)	4 (36)	59 (35)	29 (41)	76 (35)	11 (61)	80 (39)	7 (23)	76 (35)	11 (52)
Hypertension ^#ԑ^, *n* (%)																
Yes	119 (56)	28 (65)	144 (59)	3 (37.5)	132 (57)	15 (62.5)	137 (56)	10 (91)	83 (50)	44 (63)	114 (52)	12 (67)	104 (50)	22 (71)	116 (54)	10 (48)
No	92 (44)	15 (35)	102 (41)	5 (62.5)	98 (43)	9 (37.5)	106 (44)	1 (9)	83 (50)	26 (37)	103 (47)	6 (33)	100 (49)	9 (29)	98 (46)	11 (52)
Smoking status ^#ԑ^^, *n* (%)																
Past smokers	80 (38)	19 (44)	97 (39)	2 (25)	89 (39)	10 (42)	92 (38)	7 (64)	85 (51)	34 (49)	106 (49)	13 (72)	106 (52)	13 (42)	111 (52)	8 (38)
Never smokers	109 (52)	21 (49)	125 (51)	5 (62.5)	118 (51)	12 (50)	126 (52)	4 (36)	67 (40)	32 (46)	94 (43)	4 (22)	81 (40)	17 (55)	87 (40)	11 (52)
Current smokers	22 (10)	3 (7)	24 (10)	1 (12.5)	23 (10)	2 (8)	25 (10)	0 (0)	15 (9)	3 (4)	17 (8)	1 (6)	17 (8)	1 (3)	17 (8)	1 (5)
Diabetes ^#ԑ^, *n* (%)																
Yes	49 (23)	8 (19)	55 (22)	2 (25)	53 (23)	4 (17)	55 (23)	2 (18)	27 (16)	12 (17)	35 (16)	3 (17)	32 (16)	5 (16)	35 (16)	3 (14)
No	161 (76)	35 (81)	190 (77)	6 (75)	176 (77)	20 (83)	187 (77)	9 (82)	140 (84)	58 (83)	183 (84)	15 (83)	173 (84)	26 (84)	180 (84)	18 (86)
Total cholesterol, mean (SD)	195(41)	192 (41)	194 (41)	203 (44)	195 (42)	193 (35)	195 (41)	180 (52)	193 (38)	196 (39)	193 (38)	200 (41)	194 (37)	192 (41)	194 (38)	198 (32)

^#^ missing 1% in some cells, ^ԑ^ missing < 1% in some cells, ^ missing up to 5% in some cells.

**Table 3:** Multivariable analysis of the association of race and geographic region of residence with CMB.

Variable	Model	Any CMB (*n* = 213 of 808 Participants) OR (95% CI)	Non-Lobar CMB Only OR (95% CI) (*n* = 50 of 806 Participants)	Lobar CMB Only OR (95% CI) (*n* =103 of 806 Participants)	Mixed CMB OR (95% CI) (*n* = 59 of 806 Participants)	CMB Count RR (95% CI) (*n* = 213 of 808 Participants)
Overall sample						
RACE						
Black participants (Reference: White participants)	1	1.68 (1.21, 2.33)	1.51 (0.83, 2.71)	1.56 (1.02, 2.39)	1.40 (0.81, 2.41)	1.13 (0.74, 1.73)
	2	1.72 (1.22, 2.43)	1.38 (0.74, 2.56)	1.50 (0.96, 2.35)	1.68 (0.94, 3.00)	1.37 (0.88, 2.12)
REGION						
Stroke belt (Reference: non-stroke belt)	1	0.52 (0.38, 0.72)	0.44(0.23, 0.81)	0.62 (0.40, 0.95)	0.65 (0.37, 1.12)	0.91 (0.60, 1.39)
	2	0.51 (0.37, 0.72)	0.46 (0.24, 0.85)	0.60 (0.38, 0.93)	0.65 (0.37, 1.15)	0.66 (0.43, 0.99)
REGION SPECIFIC						
Stroke belt						
Black participants (Reference: White participants)	1	1.68 (1.02, 2.78)	1.79 (0.63, 5.05)	1.12 (0.56, 2.20)	2.24 (0.96,5.34)	1.32 (0.62, 2.78)
	2	1.88 (1.11, 3.20)	1.75 (0.60, 5.12)	1.19 (0.58, 2.41)	2.99 (1.19, 7.74)	1.85 (0.91, 3.80)
Non-stroke belt						
Black participants (Reference: White participants)	1	1.54 (1.00, 2.40)	1.19 (0.57, 2.48)	1.89 (1.07, 3.33)	0.95 (0.45, 1.95)	1.23 (0.75, 2.01)
	2	1.46 (0.91. 2.34)	1.21 (0.54, 2.65)	1.63 (0.89, 2.98)	1.03 (0.47, 2.22)	1.27 (0.76, 2.13)
RACE SPECIFIC						
Black						
Stroke belt (Reference: non-stroke belt)	1	0.56 (0.34, 0.91)	0.57 (0.22, 1.38)	0.50 (0.25, 0.96)	0.94 (0.41, 2.14)	0.66 (0.37, 1.17)
	2	0.56(0.33, 0.93)	0.56 (0.21, 1.38)	0.51 (0.26, 1.00)	0.94 (0.41, 2.14)	0.62 (0.35, 1.10)
Whites						
Stroke belt (reference: non-stroke belt)	1	0.52 (0.33, 0.80)	0.38 (0.15, 0.88)	0.79 (0.44, 1.41)	0.46 (0.21, 0.98)	1.06 (0.59, 1.92)
	2	0.51 (0.32, 0.80)	0.41 (0.16, 0.95)	0.75 (0.41, 1.35)	0.47 (0.20, 1.04)	0.46 (0.26, 0.84)

Model 1 adjusted for age and sex, Model 2 adjusted for age, sex, hypertension, diabetes, smoking, and total cholesterol.

## Data Availability

This study used data from the REGARDS cohort. To abide by its obligations with NIH/National Institute of Neurological Disorders and Stroke and the Institutional Review Board of the University of Alabama at Birmingham, REGARDS facilitates data sharing through formal data use agreements. Any investigator is welcome to access the REGARDS data through this process. Requests for data access may be sent to regardsadmin@uab.edu.

## Supplementary Materials

The following supporting information can be downloaded at: https://www.mdpi.com/article/10.3390/brainsci16090934/s1, Table S1: Distribution of participants with Any CMB, Non-lobar CMB, Lobar CMB and Mixed CMB. Table S2: Comparison of included and excluded participants stratified by region of residence and race. Table S3: Multivariable analysis of the association of race and geographic region of residence with CMB.
