# Supplementary material for: Race and Regional Differences in Cerebral Microbleeds in Individuals with Incident Stroke or Transient Ischemic Attack: The REGARDS Study
Source: Brain Sci. 2026 Aug 31;16(9):934. doi: 10.3390/brainsci16090934 (PMC13604854; doi:10.3390/brainsci16090934)
Supplement: Supplementary file 1 [file brainsci-16-00934-s001.zip › brainsci-4448528-supplementary.pdf]

Supplementary results

Supplementary Table S1: Distribution of participants with Any CMB, Non-lobar CMB, Lobar CMB and Mixed CMB

Demographic	Number of participants	Any CMB		Number of participants	Non-lobar CMB		Lobar CMB		Mixed CMB	
		yes	no		yes	no	yes	no	yes	no
All participants, n (%)	808	213 (26)	595 (74)	806	50 (6)	756 (94)	103 (13)	703 (87)	59 (7)	747 (93)
Black participants, n (%)	317	100 (32)	217 (68)	316	24 (8)	292 (92)	48 (15)	268 (85)	27 (9)	289 (91)
White participants, n (%)	491	113 (23)	378 (77)	490	26 (5)	464 (95)	55 (11)	435 (89)	32 (6)	458 (94)
Stroke belt residency, n (%)	412	81 (20)	331 (80)	411	16 (4)	395 (96)	40 (10)	371 (90)	24 (6)	387 (94)
Black participants, n (%)	158	38 (24)	120 (76)	157	8 (5)	149 (95)	16 (10)	141 (90)	13 (8)	144 (92)
White participants, n (%)	254	43 (17)	211 (83)	254	8 (3)	246 (97)	24 (9)	230 (91)	11 (4)	243 (96)
Non-stroke belt residency, n (%)	396	132 (33)	264 (67)	395	34 (9)	361 (91)	63 (16)	333 (84)	35 (9)	360 (91)
Black participants, n (%)	159	62 (39)	97 (61)	159	16 (10)	143 (90)	32 (20)	127 (80)	14 (9)	145 (91)
White participants, n (%)	237	70 (30)	167 (70)	236	18 (8)	218 (92)	31 (13)	205 (87)	21 (9)	215 (91)

Supplementary Table S2 Comparison of included and excluded participants stratified by region of residence and race

Variables	Region of Residence						Race					
	Stroke belt			Non-stroke belt			Black Participants			White Participants		
	Included	Excluded	P value	Included	Excluded	P value	Included	Excluded	P value	Included	Excluded	P value
Age, years mean (SD)	65 (8)	69 (9)	<0.001	68 (8)	69 (9)	0.04	66 (8)	67 (9)	0.025	67 (8)	70 (9)	<0.001
Men, *n* (%)	181 (44)	389 (47)	0.269	216 (55)	324 (50)	0.197	128 (40)	216 (38)	0.592	269 (55)	497 (55)	0.937
Women, *n* (%)	231 (56)	431 (53)		180 (45)	321 (50)		189 (60)	348 (62)		222 (45)	404 (45)	
Hypertension[#ε], *n* (%)												
Yes	263 (64)	572 (70)	0.038	252 (64)	452 (70)	0.035	241 (76)	460 (82)	0.067	274 (56)	564 (63)	0.013
No	148 (36)	245 (30)		143 (36)	191 (30)		75 (24)	103 (18)		216 (44)	333 (37)	
Smoking status [ε]												
Past	159 (39)	347 (42)	0.250	178 (45)	300 (47)	0.160	119 (38)	231 (41)	0.516	218 (44)	416 (46)	0.041
Current	58 (14)	124 (15)		39 (10)	84 (13)		54 (17)	96 (17)		43 (9)	112 (12)	
Never	195 (47)	346 (42)		178 (46)	258 (40)		144 (45)	234 (41)		229 (47)	370 (41)	
Diabetes[ε]												
yes	117 (28)	250 (30)	0.489	95 (24)	182 (28)	0.147	116 (37)	222 (39)	0.456	96 (20)	210 (23)	0.118
no	294 (71)	568 (69)		300 (76)	460 (71)		200 (63)	340 (60)		394 (80)	688 (77)	
Total cholesterol, mean (SD)	195 (43)	190 (42)	0.041	195 (40)	191 (39)	0.185	196 (45)	194 (42)	0.491	194 (39)	188 (39)	0.009

[#]missing 1% in some cells, [ε] missing <1% in some cells

Supplementary Table S3: Multivariable analysis of the association of race and geographic region of residence with CMB

variable	Model	Any CMB (*n* = 213 of 808 participants) OR (95% CI)	Non-lobar CMB only OR (95% CI) (*n* = 50 of 806 participants)	Lobar CMB only OR (95% CI) (*n* =103 of 806 participants)	Mixed CMB OR (95% CI) (*n* = 59 of 806 participants)	CMB count RR (95% CI) (*n* = 213 of 808 participants)
Overall sample						
RACE						
Black participants (Reference White participants)	1	1.72 (1.22, 2.43)	1.62 (0.87, 3.00)	1.48 (0.94, 2.31)	1.49 (0.84, 2.62)	1.37 (0.89, 2.10)
	2	1.74 (1.22, 2.50)	1.44 (0.75, 2.73)	1.44 (0.90, 2.30)	1.74 (0.96, 3.15)	1.60 (1.02, 2.47)
REGION						
Stroke belt (Reference non-stroke belt)	1	0.50 (0.36, 0.70)	0.44 (0.23, 0.82)	0.59 (0.38, 0.91)	0.65 (0.37, 1.13)	0.84 (0.56, 1.27)
	2	0.49 (0.35, 0.69)	0.45 (0.23, 0.85)	0.56 (0.35, 0.88)	0.65 (0.36, 1.14)	0.66 (0.44, 0.99)
REGION SPECIFIC						
Stroke belt						
Black participants (Reference: White participants)	1	1.59 (0.93, 2.73)	1.64 (0.54, 4.88)	1.04 (0.49, 2.14)	2.17 (0.87, 5.47)	1.71 (0.80, 3.69)
	2	1.72 (0.98, 3.02)	1.61 (0.521, 4.93)	1.09 (0.50, 2.29)	2.69 (1.01, 7.34)	2.24 (1.07, 4.69)
Non-stroke belt						
Black participants (Reference: White participants)	1	1.65 (1.04, 2.62)	1.22 (0.55, 2.66)	1.88 (1.04, 3.42)	1.10 (0.51, 2.31)	1.35 (0.82, 2.23)
	2	1.54 (0.94, 2.52)	1.21 (0.52, 2.78)	1.62 (0.86, 3.05)	1.19 (0.53, 2.61)	1.37 (0.82, 2.31)
RACE SPECIFIC						
Black						
Stroke belt (Reference: non-stroke belt)	1	0.54 (0.32, 0.90)	0.55 (0.21, 1.37)	0.49 (0.24, 0.95)	0.97 (0.41, 2.25)	0.78 (0.44, 1.37)
	2	0.51 (0.30, 0.87)	0.51 (0.19, 1.32)	0.48 (0.23, 0.96)	0.92 (0.39, 2.17)	0.74 (0.42, 1.32)
Whites						
Stroke belt (reference: non-stroke belt)	1	0.53 (0.33, 0.83)	0.41 (0.16, 0.98)	0.78 (0.43, 1.43)	0.48 (0.21, 1.02)	0.94 (0.52, 1.70)
	2	0.52 (0.32, 0.83)	0.43 (0.17, 1.04)	0.74 (0.39, 1.36)	0.49 (0.21, 1.10)	0.57 (0.32, 1.02)

MODEL 1: Adjusted for age, sex and MRI sequence. Model 2: adjusted for age, sex, MRI sequence, hypertension, diabetes, smoking, and total cholesterol
